# Content analysis of identity challenges in patients with haemophilia: A qualitative study

**DOI:** 10.1002/nop2.761

**Published:** 2021-01-06

**Authors:** Roya Dolatkhah, Reza Shabanloei, Hossein Ebrahimi, Mostafa Ghasempour

**Affiliations:** ^1^ Hematology and Oncology Research Center Tabriz University of Medical Sciences Tabriz Iran; ^2^ Department of Medical—Surgical Nursing Nursing and Midwifery Faculty, Sina Educational, Research and Treatment Center Tabriz University of Medical Sciences Tabriz Iran; ^3^ Department of Psychiatric Nursing, Nursing and Midwifery Faculty Tabriz University of Medical Sciences Tabriz Iran

**Keywords:** challenge, content analysis, identity, nurses, nursing, qualitative study

## Abstract

**Aims:**

Inherited bleeding diseases greatly affect education, working, job, social activities and quality of life. We aimed to discover the sources of identity challenges among patients with Haemophilia.

**Design:**

The present study has been designed and performed as a qualitative content analysis research.

**Methods:**

Participants were purposively selected from haemophilia patients referred to our clinic during one year, from March 2018 to April 2019. Data collection was done through semi‐structured, in‐depth interviews using purposeful sampling. Data were analysed based on Granheme and Landman method. The main categories were fear of rejection, losing social roles, discrimination and stigma and marriage breakdown.

**Results:**

Patients with haemophilia encounter several challenges due to physical and social constraints caused by the disease. Such challenges result in disturbances in the self‐identity of the patients.

**Conclusion:**

According to the results of this study, the nurses should plan to have attention to the patients with haemophilia, based on more protection and better supports.

## 1 INTRODUCTION

Haemophilia is a heredity bleeding disease which is more common among males (Stonebraker et al., 2020). Regarding the spread of this disease, Iran holds 9th global rank and is the second among Mediterranean area (Dolatkhah et al., 2014; Dorgalaleh et al., 2016). Improvement of healthcare standards of patients with haemophilia, such as using reintegration factor concentrates greatly enhanced the prevention and treatment of blood shedding and has led to promote patients' quality of life (QoL) and the disease's endurance (Carroll et al., 2019; Davari et al., 2019; Singh et al., 2020; Stonebraker et al., 2020; Trindade et al., 2019; Usuba et al., 2019). However, bleeding disease has affected their education, job, social activities and family life tremendously. Patients with haemophilia encounter many clinical and psychological challenges that can cause emotional diseases. Such factors can have effect on the acceptance of the disease and patients' health. Therefore, to achieve a comprehensive nursing care among the individuals with haemophilia, there is a need to pay more attention to psychological and social health of the patients (Pinto et al., 2018).

### 1.1 Background

The change of lifestyle from an active to a passive can cause the loss of social relationships, and the reduction of economic status due to the limitations caused by the disease can change the identity of patients. Having stressful experiences from family members and workplace may lead to negative response towards the disease and its outcomes (Davari et al., 2019). Chronic diseases may affect the person's identity and his/her life route deeply (Catchpole & Garip, 2019). Therefore, the effect of the disease and the outcomes caused by it should be considered more seriously regarding the identity of a patient. Thus, it is necessary for the healthcare, the relatives and friends of the patients to understand the passing changes of the patients and take care of them (Cruwys et al., 2020). To some extent, the biographical disruption and lifestyle changes experienced by patients could imply that their previous sense of identity became more or less invalid (Larun & Malterud, 2007). Identity is a concept that can be considered as a biography chain of incidents and the nucleus of an individual's identity hidden within his/her capability to continue narration (Giddens, 1991). Pierce et al. believed that individuals are enthusiastic to be praised and to present their identity (Pierce et al., 2003). Self‐identity is also the borderline between an individual and the society in a way that an individual develops self‐identity feelings through observing himself/herself by using a social perspective (Batra et al., 2016).

Therefore, identification is a dynamic state and a conclusion of the reflection of a person regarding the relations with others and the definition of “ego” interpreted, although he/she is with a chronic disease or being a patient (Karnilowicz, 2011). It seems that the deeper understanding of the way through which the patient with haemophilia encounters identity challenges will present an important knowledge base for the healthcare specialists and others dealing with the patients (Reinicke et al., 2019). Thus, the aim of present study was to describe possible sources of identity challenges among male patients with haemophilia referred to Shahid Ghazi Hospital, Hematology and Oncology Research Center of Tabriz. As haemophilia patients had many psychological problems, that altered their quality of life and behaviours, the present study was qualitative content analysis research, including the process of perception, interpretation and conceptualization of the internal meanings of qualitative data.

## 2 METHODS

### 2.1 Design

The present study designed and performed as a qualitative content analysis research, during one year from March 2018–April 2019, at Hemophilia Clinic of Shahid Ghazi Hospital, Tabriz, Iran. This research method entails the process of perception, interpretation and conceptualization of the internal meanings of qualitative data (Rebar & Macnee, 2010). Considering the research aims, the participants were purposively selected from patients with haemophilia referred to our clinic to receive concentrated products and factor VIII and/or to receive routine healthcare facilities. The inclusion criteria for this study were as follows: patients with severe haemophilia A disease, permanent need to receive concentrated factors, ages between 18–50, consciousness and desire to express feelings towards the study concept. All participants were male due to gender dependence. Five participants were employee, three participants were self‐employed, three patients were part‐time worker, two participants were unemployed, and one participant was student.

### 2.2 Method

To collect data and saturation of them, semi‐structural face‐to‐face interview has been performed by RS of 14 participants, who referred the clinic consecutively during the time of study. This type of interview is appropriate for qualitative studies due to its flexibility and in‐depth features (Speziale et al., 2011). The general questions were devised as interview guide for the interview that led the interviewee to probing questions to clarify the issue. The interviews were conducted in a quiet and undisturbed room at the haemophilia clinic so that the participant could concentrate, and their voices were recorded safely. The duration of interviews was between 25–45 min.

Based on the Qualitative Studies interview method, the interview began with a general question, for example: “When your diseases has been diagnosed?”, “What were your first symptoms?” Then, continued with the key questions, for example: “What problems has your diseases caused you?” Probing questions were asked to clarify the issue for example: “What you mean by *the disease has darkened my life*? Can you explain more?” If he had theoretical questions from previous interviews, the researcher asked the next participant directly. For example, according to one previously participant statement, the researcher asked this question from next one: “One of Haemophilia patients said that: ‘My disease caused my wife to file for divorce’. Did you have the same experience? How did you challenge with this?” Finally, participants were asked to add for any additional questions or unclear aspects of interview. We excluded five patients, who were not meet our inclusion criteria. Interviews and their analysis were constantly reviewed by co‐researchers (RD, HE) who were qualitative researchers. Main categories and subcategories of studied identity challenge in haemophilia patients were presented in Table 1.

**TABLE 1:** Identity challenge in haemophilia patients: categories and subcategories

	Main categories	Subcategories
Identity challenges	Fear of rejection	Preference of disease secrecy upon health
		Colleagues' dissatisfaction with the patient's absence from work
		Family dissatisfaction with wasted time to treating the patient
		Feeling imposed on the family
	Fear of losing social roles	Numerous experiences of defeat due to illness
		Loss of dreams due to illness
		Decreased quality of life due to illness
		Loss of family roles due to illness
		Loss of social roles due to illness
	Fear of discrimination and stigma	Experiencing disease stigma The stigma of contagious disease The stigma of being vulnerable The stigma of hereditary transmission of disease
		Restrictions of independence
		Fragility of society due to patient constraints
	Fear of marriage breakdown	Defects in the role of spouse
		Family challenge due to medical abortion
		Sexually and emotionally challenges affected by Disease
		Economic Challenges affected by Disease

### 2.3 Analysis

The data analysis was performed based on the method proposed by Graneheim and Lundman (2004). Immediately after each interview, audio‐recorded interviews were listened to and were transcribed. Each transcribed interview was read again and again to give a whole recognition of the content. All the interviews were divided into meaning units that were condensed into a description close to the text, abstracted and labelled with a code. All the codes extracted from the interviews were reviewed by the researchers, and after agreement among the members of the research team, they sorted into subcategories and categories. Then, all codes were formed and compared based on differences and similarities.

### 2.4 Rigour

The most important criteria to determine rigour or trustworthiness in a qualitative study are credibility, dependability, conformability and transferability (Rebar & Macnee, 2010). In this study to enhance validity and reliability, we used the criteria suggested by Lincoln and Guba (1985). For credibility, the researcher spent enough time in setting for data collection. To ensure data precision, the researcher used member check in addition to bracketing of previous preconceptions. For achieving reliability, along with the recording and documentation of the interviews, the very sheer expressions used by the participants were used to reinvestigate them if needed. To approve coding steps and forming the categories and subcategories, the researcher used peer check. For transferability, the researcher tried to choose the samples with most different varieties:

- Sufficient time spent by the researcher allows the researcher to carefully observe and examine the behaviours of the participants.
- The researcher's immersion in the data during the research period causes the researcher to have a deep understanding of the concepts.
- Returning to the participants to confirm the findings will help the participants to critique the findings and improve its quality and variability.
- For transferability, some information was included about patients' mean age, education levels, economic status and patients' physical problems caused by haemophilia.

### 2.5 Ethics

This study has been accepted and supported as research project in Hematology and Oncology Research Center (Code: 90/14:90/9/23) and was approved by the ethics committee of the University (ID: 3066:90/11/6). All the participants were informed about the process, study method and the ability to withdraw from participation whenever they wished. Also, the patients' data were kept confidential and signed consent forms were obtained.

## 3 RESULTS

Haemophilia patients were not only challenged with aspects of their disease, but psychological problems also have a negative impact on their lifestyle. So that, these patients can feel their personal identity has been affected by their disease. Thus, these patients suffer from tension and stress and lose the comfort in their life. Fear is recognized as one of the major aspects of the problems among such patients. Among these patients, fear of identity problems is much more important than fear of physical status caused by the disease. The responses given by the participants were categorized into four main categories: fear of rejection, fear of losing social roles, fear of stigma and discrimination and fear of marriage breakdown.

### 3.1 Fear of rejection

Regarding that there may be incorrect perceptions about haemophilia disease, the participants have claimed that they prefer hiding their disease. A participant said:

It's impossible to tell people that I am a haemophilic patient. Especially in public places, although there is the risk of getting hit, falling on the ground and getting hurt. And if you tell them, they will do nothing for you. They will only try to avoid you (P3).

Also, the urgent needs of such patient are to go to a hospital to receive concentrated factors that usually takes several hours will irritate the colleagues and sometimes their family members. A participant said:

Often you are forced to stop working and look for Factor VIII or other concentrated products and it takes several hours. In this case, your colleagues should pick up the load. They can't do it all the time. Gradually they and the boss will complain (P7).

Another patient said:

Sometimes you are forced to tell them [your family] that I won't join them because there isn't any factor at home. Or sometimes your knee is bleeding and you should stay at home several days vulnerably. In this case, gradually the family will complain and say what a life you have created for them (P8).

The feeling of being a burden on the others and the inability in performing their role, specifically in the family will encourage a weak and vulnerable identity to the patient. A participant said:

Most of the times I feel that I am not in charge, but a burden on the family (P1).

Therefore, these patients feel that their identity will be endangered if they are not accepted by the community and family.

### 3.2 Fear of losing social roles

Experiencing defeats or losing the wishes due to disease and its complications has caused most patients not enough incentive to create new opportunities during their life and this will result in reducing the quality of life for the patients. A patient said:

In fact, patients with Haemophilia live at the moment; they can't plan anything for even near future because they may fall today or may get hurt and stay in bed for few days (P4).

Physical constrains and therapeutic needs of patients with haemophilia create problems with their social roles and even family roles. Losing roles leads to low self‐esteem among patients. A patient said:

When you lose your job you lose your identity. If you are an engineer (patient was an engineer) but you lose your job [because of your disease], you are not the one that you have been before (P1).

So loss of social roles is one of the factors that cause a sense of instability in the existential identity of these patients.

### 3.3 Fear of discrimination and Stigma

These patients have noted that lack of acceptance by the society is due to the mark and stigma caused by the disease or the probability of the patient causing harm to the others. A patient said:

Most people think that Haemophilia is transmissible disease like AIDS or due to frequent injections they think we also have infectious diseases (P2).

Since haemophilia type A is a disease depending on gender and can be transferred or inherited, most patients report multiple rejections of their marriage. A patient said:

My classmate and I were going to get married and even though we had gone through genetic counseling, her family didn't approve our marriage and we departed (P5).

Another patient was crying hard and saying: “*They [*in‐laws*] told my wife that he is sick. At any moment he can bleed and die and you will become a widow and you have to raise his orphans (P1*).”

The dangers caused by bleeding have led most family members to approach the patient cautiously and banish them from doing some chores. These cautious behaviours of family members lead to inspire weakness or loss of value of the patients. A patient said:

They treat me like a baby. If there is some snow, my mother doesn't let me go out. She doesn't let me touch the knife. She has put some blankets around my bed, as if I am a child and I don't know my weaknesses and strengths (P6).

Several experiences of stigma by the society or the lack of acceptance by the society have discouraged more patients to ask for help and to accept living with torturous condition.

When a 35 years old patient said about asking the colleagues for help in workplace:

I don't ask for help much. The health workers told me several times, if you are sick you should ask for early retirement and pension. Now I feel they can't stand me. I try to do everything by myself, although sometimes I feel lots of pressure (P11).

Experiences of stigma, discrimination and disrespect, whether due to illness or unnecessary pity, cause the patient to feel hopelessness and incompatibility.

### 3.4 Fear of marriage breakdown

Physical movement constraints specifically in knee joints may cause problems in performance and sometimes patient feels distressed. A patient said:

Before marriage my knee didn't have much problem. But gradually I lost my knee joints and now I‘m limping. Meanwhile, I think my wife is ashamed of my condition in front of her relatives and friends and on the other hand I can't afford the life for my family (P9).

Because of the possibility of hereditary transmission of haemophilia A disease in female foetus, in the case of the pregnancy of the spouse to the female baby, the couple try to abort after genetic tests showing that their baby is a carrier and this causes some challenges among their families. A patient said:

Once my wife was pregnant and the baby was a girl. She went to Tehran for a genetic test. Finally, they said that the fetus is a carrier. She asked to abort the fetus without my consent. After abortion, she could not be pregnant for 3 to 4 years. For that reason, we were even about to get divorced (P10).

The probability of being infected by blood transmitted infectious diseases, not only caused marks in such patients, but also these patients lose most of their roles. A patient said:

I suffer from Hepatitis C because of blood products’ transfusions. Ever since I realized that I have been infected by a disease (about 12 years ago), my wife decided to get divorce but her father didn't agree. Since then, we have never had any sexual and intimate relationships. Even our only child is raised in his grandparents' house. He rarely comes to see me. He doesn't accept me as his father (P12).

One of the problems that married haemophilia patients have is their inability in establishing a satisfactory sexual relationship with their spouse and lack of satisfaction of their spouse is caused by the physical problems with such patients. A patient said:

The sexual relationship of the patients with Haemophilia is symbolic. Not only you don't enjoy, but also your wife does not enjoy. There is not any articulation to enjoy (P10).

Physical limitations and job and income constraints among patients with haemophilia lead to the financial restrictions of patients and their families. Such difficult situation in life may sometimes accompany the patient's inability in affording the life and this may sometimes cause verbal quarrels and dissatisfaction in the family. A patient said:

My wife says you have neither good moods nor income. What am I happy about you? (P12).

Deficiencies in family role‐playing and inducing the burden of being a family burden because the patient to worry about the marital status is being disturbed.

## 4 DISCUSSION

According to the results of the present study, patients with haemophilia encounter several challenges due to physical and social constrains caused by the disease. Such challenges result in disturbances in self‐identity of the patients. In this study, all participants proposed that one of the principal items in creation of identity challenges is constraints in physical activities. In a similar study by Asbring et al. carried out among women with chronic fatigue syndrome and fibromyalgia, it was concluded that constraints in work and social life are among very important factors dominating the patients' identity. Meanwhile, the experience of the disease may cause some positive changes in identity due to a new outlook regarding the life (Asbring, 2001). This contradiction may depend on gender and age of the participants in the two studies because men are more sensitive to their identity than women and losing their identity is more harmful (Catchpole & Garip, 2019). In a qualitative study by Zeinalian et al. (2014) performed on patients with haemophilia, the fear of being isolated and repelled by the society has caused the patients to avoid revealing their disease. The history of frequent defeats caused by the disease has led patients with haemophilia to encounter the fear of losing their social role.

In addition to clinical problems, patients with haemophilia encounter psychological challenges such as doubt, social limitations and losing their jobs (Iannone et al., 2012; Pinto et al., 2018). Due to poor self‐identity, financial problems, chronic pain and drug abuse, suicide is frequently reported in haemophilia (Fakhari & Dolatkhah, 2019). In a study by Ghanizadeh & Baligh‐Jahromi (2009), children and adolescents with haemophilia suffered stress problems and 36% of them prefer death to life and 6% of them think of committing suicide . Although our participants did not report any suicidal thoughts, losing their social roles was claimed to be considered as a defeat in identity. This difference may have been caused by the increased ability to cope with the disease among the adults as time passes.

In this study, the mark of being sick has been claimed to be one of the principal challenges among the patients. Studies carried out on patients with haemophilia showed that stigma is one of the main concerns of such patients and they challenge to hide the disease because they had fear of revealing their disease and encountering discrimination (Boardman et al., 2019; Reinicke et al., 2019). In a study by Barlow et al. (2007), one of the most important problems of patients with haemophilia was stigma and discrimination due to incorrect perception of the society from infectious diseases (such as HIV and HCV) and the patients' vulnerability. In the present research, in addition to the experience of stigma by the society, the patients suffered from doubts in their personal identity due to the experience of stigma and discriminated by the family because of their poor understanding of this disease. Although the protection of the patients by the family, specifically parents were considered as spiritual, psychological and physical support, some studies indicate overly concerned behaviour of family can cause weakness and inability among the patients (Zeinalian et al., 2014).

In a study by Semple et al. (2008), patients with head and neck cancers had claimed that the reduction of their self‐esteem and being disappointed with the future caused them to avoid socializing and this may have caused them to lose their personal identity, self‐esteem or roles. In another research study carried out by Rambod et al. (2016), chronic and severe pains were introduced as the factors causing sadness and psychological distress among patients with haemophilia that could affect their social activities and change their relationships. In this study, also the patients who were disappointed with the future considered to have lost their self‐identity and their roles.

Sexual problems are among the basic problems of the patients with haemophilia. These patients have problems in erection and ejection due to physical pain and joint problems and psychological problems such as stress, mark and the probability of transferring infectious diseases and the attitudes of the family and the society and different medical treatments. These factors harm sexual health of the person (Blamey et al., 2019). Hassani et al. performed a research study on Chronic Hepatitis B Patients. They stated that the lack of tolerance among the spouses, lack of success in married life, lack of understanding the nature of the disease by the spouse, high costs of self‐care, being disappointed by life partner's blames, being shamed by spouses and hiding the disease from family members and fear of marriage failure are the main causes of failed relationship between the couples (Hassani et al., 2017). Men encounter several identity and social challenges due to bleeding diseases caused by several inabilities that cause lack of response to the traditional expectations of the society from men such as performing the roles of being a father, a husband and a breadwinner. Thus, behaviours that cause the loss or reduction of self‐esteem and feeling of downgrade will cause identity problems and low self‐respect towards the individual himself (Gallagher et al., 2008).

## 5 CONCLUSION

Adult patients with haemophilia cannot perform many of the roles that society and family expect to do due to their physical status and dangers caused by bleeding in Iran. Even in a worse situation, men lose many of their roles. On the other hand, psychological problems caused by the disease and the experience of stigma and frequent discriminations have led these patients to feel distressed and lose their self‐esteem and finally change their personal identity. This study has identified the challenging aspects of these issues, and it is expected that shedding light on them can alleviate the problems that patients with haemophilia have.

### 5.1 Research limitations

This study has been carried out using a qualitative method in Iran. Therefore, it may not be generalizable in other contexts and cultures.

## CONFLICT OF INTEREST

The authors stated that they had no interests which might be perceived as posing a conflict or bias.

## AUTHOR CONTRIBUTIONS

RD and RS made substantial contributions to the conception or design of the work; the acquisition, analysis or interpretation of data; HE and MG drafted the work and revised it critically for important intellectual content; RD, RS, HE and MG approved the version to be published; and All the authors agree to be accountable for all aspects of the work in ensuring that questions related to the accuracy or integrity of any part of the work are appropriately investigated and resolved.

## Data Availability

Researchers allow this article to be used by anyone interested.
